# Investigating hookworm genomes by comparative analysis of two *Ancylostoma *species

**DOI:** 10.1186/1471-2164-6-58

**Published:** 2005-04-26

**Authors:** Makedonka Mitreva, James P McCarter, Prema Arasu, John Hawdon, John Martin, Mike Dante, Todd Wylie, Jian Xu, Jason E Stajich, Wadim Kapulkin, Sandra W Clifton, Robert H Waterston, Richard K Wilson

**Affiliations:** 1 Genome Sequencing Center, Department of Genetics, Washington University School of Medicine, St. Louis, MO 63108, USA; 2 Divergence Inc., St. Louis, MO 63141, USA; 3 College of Veterinary Medicine, Department of Molecular Biomedical Sciences, North Carolina State University, Raleigh, NC 27606, USA; 4 Department of Microbiology and Tropical Medicine, George Washington University Medical Center, Washington, DC 20037, USA; 5 Department of Molecular Genetics and Microbiology, Duke University, Durham, NC 27710, USA; 6 Department of Infectious Diseases, Microbiology and Parasitology, Faculty of Veterinary Medicine, Warsaw Agricultural University, Warszawa, Poland; 7 School of Biology, University of Leeds, LEEDS LS2 9JT, UK; 8 Department of Genome Sciences, University of Washington, Seattle, WA 98195, USA

## Abstract

**Background:**

Hookworms, infecting over one billion people, are the mostly closely related major human parasites to the model nematode *Caenorhabditis elegans*. Applying genomics techniques to these species, we analyzed 3,840 and 3,149 genes from *Ancylostoma caninum *and *A. ceylanicum*.

**Results:**

Transcripts originated from libraries representing infective L3 larva, stimulated L3, arrested L3, and adults. Most genes are represented in single stages including abundant transcripts like hsp-20 in infective L3 and *vit-3 *in adults. Over 80% of the genes have homologs in *C. elegans*, and nearly 30% of these were with observable RNA interference phenotypes. Homologies were identified to nematode-specific and clade V specific gene families. To study the evolution of hookworm genes, 574 *A. caninum */ *A. ceylanicum *orthologs were identified, all of which were found to be under purifying selection with distribution ratios of nonsynonymous to synonymous amino acid substitutions similar to that reported for *C. elegans */ *C. briggsae *orthologs. The phylogenetic distance between *A. caninum *and *A. ceylanicum *is almost identical to that for *C. elegans */ *C. briggsae*.

**Conclusion:**

The genes discovered should substantially accelerate research toward better understanding of the parasites' basic biology as well as new therapies including vaccines and novel anthelmintics.

## Background

Comparative sequence analysis is an approach proven to aid in recognition of genes and defining of their function, especially when comparing genomes of close evolutionary distance. In addition, when partial genomes are placed in a context of a well-studied and fully sequenced model organism they can greatly facilitate the understanding of the less studied organisms' biology.

Hookworms are blood-feeding nematodes that infect one billion people causing iron deficiency anemia and retarded physical and cognitive development in children [1]. The two major species infecting humans are *Necator americanus *and *Ancylostoma duodenale*. The closely related hookworm species of canids, *Ancylostoma caninum*, and canines and felines, *A. ceylanicum*, are minor parasites of humans, but are important as laboratory models for hookworm infection and disease. Other hookworms infect raccoons, sheep, seals and a variety of other mammals [2]

Adult (Ad) hookworms inhabit the small intestine and produce eggs that pass in the feces and hatch in the soil. The first stage larva (L1) feeds on bacteria and molts twice to form the non-feeding, infective third stage (iL3). iL3 enters the host by penetrating the skin, or orally in the case of *Ancylostoma *species, molts twice, and matures to Ad in the small intestine. *A. duodenale *and *A. caninum *L3s can also infect a host, temporarily abort maturation and enter an arrested state (hypobiosis) within the host's somatic tissues [3], reactivating in response to host physiological changes such as pregnancy [4].

Current hookworm control strategies are limited to de-worming of infected people using anthelmintic drugs. However, rapid re-infection in endemic areas and the lack of sterile immunity necessitates repeated treatments and can in turn result in resistance. Additionally, tissue-arrested stages are relatively resilient to the effects of anthelmintics [5]. The Human Hookworm Vaccine Initiative is beginning clinical trials of a larval hookworm antigen, ASP-2, from *N. americanus*, as a vaccine antigen [6]. There is a critical need for further research to identify new vaccine and drug targets as well as to better understand hookworm biology. Lack of sequence information has been a major hindrance to hookworm molecular studies. High throughput sequencing of expressed sequence tags (ESTs; sequences derived from randomly selected cDNA clones) has proven a cost-effective tool for discovering genes [7]. Because the hookworm superfamily (Ancylostomatoidea) falls within nematode Clade V [8,9], which also contains the well-studied model nematode *Caenorhabditis elegans *[10], predictions may be made and tested based on their close relatedness. Previous genome-based characterization of hookworms has been limited to sampling of few hundred ESTs [11] and molecular studies of individual genes of interest (eg. [12]; reviewed in [13]). EST approaches have also been initiated for other Strongylid parasites including *Haemonchus contortus *[14,15] and *Nippostrongylus brasiliensis *[16].

In this report we describe the comparative analyses of almost 20,000 ESTs from 7 different cDNA libraries representing pre-parasitic and parasitic larval through adult stages of the hookworms *A. caninum *and *A. ceylanicum*. The dataset defined nearly 7,000 hookworm genes, including a number of putative developmentally expressed genes and candidates for further study as drug target or vaccine components.

## Results

Nearly 20,000 *Ancylostoma *derived ESTs were submitted to GenBank between 1999 and 2003 [see Additional file 1]. For simplicity, the results and analysis described are presented in the same order beginning with *A. caninum *and followed by *A. ceylanicum*, except where specified.

### EST acquisition and NemaGene organization

**Figure 1:**
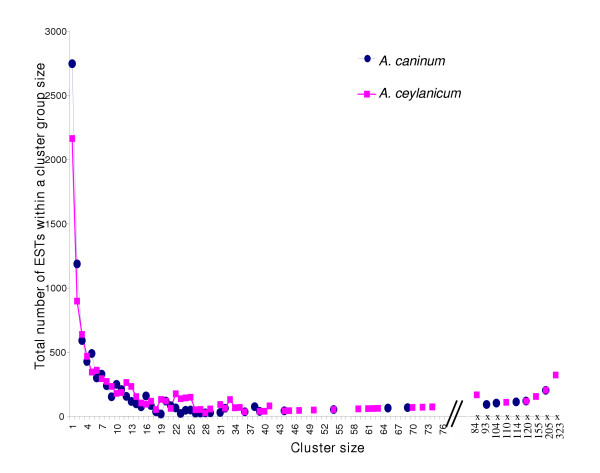
*Ancylostoma *NemaGene v2.0 clustering showing the distribution of ESTs by cluster size. For example, there are three *A. caninum *cluster of size 22 containing a sum of 66 ESTs and there were eight *A. ceylanicum *clusters of size 22 containing a sum of 176 ESTs. Cluster size (x-axis) is shown to scale for 1–75 members, with the size of larger clusters indicated.

ESTs originated from 7 cDNA libraries, representing three and two life-cycle stages respectively (Table 1). Clustering, implemented to reduce data redundancy and improve sequence quality and length, grouped ESTs into contigs which were further organized into clusters (Table 1), providing a non-redundant catalog of represented genes. ESTs within a contig derive from nearly identical transcripts while contigs within a cluster may arise from splice isoforms, alleles, or closely related paralogs [17]. Fifty-one potentially chimeric ESTs were discarded. Clusters ranged in size from a single EST to 203 and 323 for *A. caninum *and *A. ceylanicum *respectively (Figure 1). Most clusters for each species (72% and 55%) have ten or fewer ESTs. GC content for coding sequences was similar in the two species (44% and 48%) and consistent with other Clade V nematodes like *C. elegans *and *C. briggsae *[18].

**Table 1:** *Ancylostoma *libraries sequenced and their properties

	Nematode stage (vector or SL1 based)	ESTs Submitted	Nucleotides (million)	Mean read length (bp)	StDev
*A. caninum*	Infective L3 (UniZap)	5,679	2,632	358	109
	Tissue arrested L3 (SL1)	820	0,318	344	151
	Serum stimulated L3 (pAMP)	2,832	1,273	441	150
	Overall	9,331	4,223	381	137
					
	Contigs – 5,484 (4,020 clusters)			502	168

*A. ceylanicum*	Infective L3 (λZAP II)	3,359	2,021	500	127
	Infective L3 (SL1)	3,306	1,550	400	143
	Adult (M1 SL1)	629	0,319	460	134
	Adult (M2 SL1)	480	0,255	467	131
	Adult (λZAP II)	2,817	1,646	500	139
	Overall	10,591	5,791	465	135
					
	Contigs – 4,953 (3,369 clusters)			572	179

**Figure 2:**
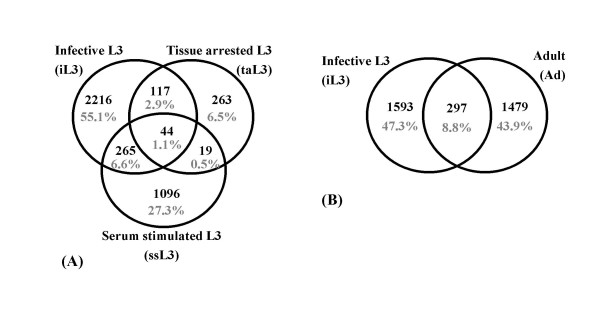
Venn diagram of *A. caninum *(A) and *A. ceylanicum *(B) clusters, based on stage of origin of each cluster's EST members. The majority of clusters are represented by only one stage in this investigation, though greater depth of sampling would likely increase representation by multiple stages.

The number of clusters may overestimate gene discovery, as one gene may be represented by multiple non-overlapping clusters (fragmentation). By using *C. elegans *as a reference genome (19,522 genes; [10]) and discounting for fragmentation calculated as 4.5% and 6.5 % respectively [17], the estimated gene numbers were reduced to 3,840 for *A. caninum *and 3,149 for *A. ceylanicum *giving a gene discovery rate of 41% (3,840 × 100/9,283) and 30% (3,149 × 100/10,588). These numbers also indicate 20% and 16% representation of all genes for each species respectively. The number of genes in common for more stage-specific *Ancylostoma *libraries analysed was as low as 9% and 11% (Figure 2). This may reflect the EST sample size or stage-specific expression, as will be discussed. In either case, the results clearly show the advantage gained for gene discovery in *Ancylostoma *by including diverse life stages in the analysis.

### Functional classification based on Gene Ontology and KEGG assignments

Thirty-four percent of *A. caninum *and 54% of *A. ceylanicum *clusters align to InterPro domains and 21% and 36% map to Gene Ontology (GO) respectively. Following this same pattern, *A. caninum *also had fewer BLAST matches (see below). Seven of the ten most abundantly represented InterPro domains were common to both species (Table 2). GO representation is shown by biological process, cellular component, and molecular function (Table 3). Among the most common GO categories are protein metabolism (GO:0019538) and catalytic activity (GO:0003824).

**Table 2:** Most abundantly represented protein domains in *A. caninum *and *A. ceylanicum *datasets

Species	InterPro ID	Clusters #	Domain descriptor
*A. caninum*			
	IPR001230	141	Prenyl group, CAAX box, attachment site
	IPR001687	66	ATP/GTP-binding site motif A (P-loop)
	IPR000694	62	Proline-rich region
	IPR001472	51	Bipartite nuclear localization signal
	IPR000345	29	Cytochrome c heme-binding site
	IPR001283	25	Allergen V5/Tpx-1 related
	IPR002048	24	Calcium-binding EF-hand
	IPR000504	21	RNA-binding region RNP-1 (RNA recognition motif)
	IPR000719	19	Protein kinase
	IPR007087	17	Zn-finger, C2H2 type
*A. ceylanicum*			
	IPR000694	214	Proline-rich region
	IPR001230	153	Prenyl group, CAAX box, attachment site
	IPR001687	125	ATP/GTP-binding site motif A (P-loop)
	IPR000345	50	Cytochrome c heme-binding site
	IPR006209	48	EGF-like domain
	IPR000504	37	RNA-binding region RNP-1 (RNA recognition motif)
	IPR001283	34	Allergen V5/Tpx-1 related
	IPR001472	32	Bipartite nuclear localization signal
	IPR000169	32	Eukaryotic thiol (cysteine) protease
	IPR001534	27	Transthyretin-like

**Table 3:** GO mappings for *A. caninum *and *A. ceylanicum *clusters

		*A. caninum*		*A. ceylanicum*	
Categories and subcategories		Representation	% Representation of total	Representation	% Representation of total
biological process					
cellular process		192	4.80	238	4.36
cell communication		62	1.55	66	1.21
cell motility		1	0.03	0	0.00
cell death		1	0.03	1	0.02
cell growth and/or maintaince		140	3.50	180	3.30
	transport	119	2.98	153	2.80
	cell organization and biogenesis	21	0.53	32	0.59
	cell proliferation	4	0.10	6	0.11
	cell homeostasis	1	0.03	4	0.07
physiological process		579	14.48	785	14.38
response to endogenous stimulus		1	0.03	5	0.09
response to external stimulus		12	0.30	16	0.29
response to stress		8	0.20	14	0.26
death		1	0.03	1	0.02
metabolism		466	11.65	653	11.96
hemostasis		1	0.03	0	0.00
homeostasis		3	0.08	4	0.07
secretion		0	0.00	1	0.02
development		11	0.28	18	0.33

cellular component					
cell		327	8.18	385	7.05
intracellular		212	5.30	277	5.08
	cytoplasm	166	4.15	181	3.32
	nucleus	45	1.13	84	1.54
	ribonucleoprotein complex	102	2.55	102	1.87
	respiratory chain complex	4	0.10	5	0.09
	chromosome	8	0.20	13	0.24
	thylakoid	0	0.00	1	0.02
membrane		146	3.65	146	2.67
extracellular		33	0.83	50	0.92
Unlocalized		1	0.03	7	0.13

molecular function					
binding		323	8.08	521	9.55
carbohydrate binding		6	0.15	20	0.37
lipid binding		6	0.15	12	0.22
metal ion binding		56	1.40	72	1.32
nucleic acid binding		109	2.73	201	3.68
nucleotide binding		133	3.33	214	3.92
protein binding		11	0.28	22	0.40
apoptosis regulator activity		1	0.03	1	0.02
chaperone activity		5	0.13	10	0.18
cell adhesion molecule activity		2	0.05	1	0.02
catalytic activity		293	7.33	445	8.15
enzyme regulator activity		24	0.60	35	0.64
molecular function unknown		38	0.95	46	0.84
motor activity		3	0.08	12	0.22
signal transducer activity		55	1.38	61	1.12
structural molecule activity		107	2.68	127	2.33
transcription regulator activity		19	0.48	33	0.60
translation regulator activity		14	0.35	17	0.31
transporter activity		128	3.20	180	3.30

Within *Ancylostoma spp*. clusters that had extracellular mappings, 70% and 56% respectively were in the category of Allergen V5/Tpx-1 proteins (IPR001283) related to the secreted venom proteins from hymenopteran insects (Table 2). The *Ancylostoma *secreted proteins (ASPs) belong to this large gene family [19], members of which been shown to play roles in host-parasite interactions for both mammalian [20,21] and plant parasitic nematodes [22], and to induce protective responses [6]. ASP-1 is one of the major proteins secreted by serum-stimulated *A. caninum *iL3 [12]. In addition, four *A. ceylanicum *clusters were classified in extracellular matrix (GO:0005578) as tissue inhibitor of metalloprotease (TIMP) domain proteins. A TIMP homolog is reported as the most abundant protein in adult hookworm excretory/secretory products and may inhibit host metalloproteases [23].

Ten % and 15% unique clusters for *A. caninum *and *A. ceylanicum *respectively, mapped to 89 metabolic pathways grouped in 11 categories (Table 4). Complete listings and graphical representations of the KEGG mappings are available at . Pathways well represented by both species include glycolysis/gluconeogenesis, citrate cycle, oxidative phosphorylation and fatty acid biosynthesis and metabolism. KEGG analysis (Table 4) also suggests specific biochemical differences among *Ancylostoma *stages. For example, while serum stimulated L3-specific clusters make up to 27% of all AC clusters, they account for 40% of all KEGG pathway mappings. In contrast, iL3-specific clusters that account for 55% of all AC clusters make-up only 38% of KEGG pathway mappings. It is unclear whether the predominance of enzyme mappings from the ssL3 stage versus iL3 stage is indicative of greater metabolic activity, greater metabolic complexity, differences in library construction methods, or other differences.

**Table 4:** Kegg Biochemical pathway mappings for *A. caninum *and *A. ceylanicum *clusters

	AC	Clusters per library					AE	Clusters per library				Total # of enzymes in KEGG
							
KEGG CATEGORY REPRESENTED^a^	Cl^b^	iL3	taL3	ssL3	Mixed	Enz^c^	Cl^b^	iL3	Ad	Mixed	Enz^c^	
1. Carbohydrate metabolism												
1.1 Glycolysis / Gluconeogenesis	23	13	0	5	5	22	25	10	11	4	23	40
1.2 Citrate cycle (TCA cycle)	17	8	0	4	5	16	15	4	8	3	15	23
1.3 Pentose phosphate pathway	9	3	0	4	2	9	12	5	3	4	8	34
1.4 Pentose and glucuronate interconversions	8	3	0	3	2	9	9	5	4	0	8	53
1.5 Fructose and mannose metabolism	14	6	0	4	4	15	20	7	11	2	15	61
1.6 Galactose metabolism	10	4	0	4	2	8	12	6	5	1	12	37
1.7 Ascorbate and aldarate metabolism	7	4	0	2	1	4	5	5	0	0	4	29
1.8 Pyruvate metabolism	25	11	0	11	3	23	27	8	17	2	26	67
1.9 Glyoxylate and dicarboxylate metabolism	13	6	0	5	2	14	9	1	5	3	17	58
1.10 Propanoate metabolism	22	7	1	10	4	20	25	11	8	6	22	46
1.11 Butanoate metabolism	22	9	1	7	5	23	29	14	14	1	26	52
1.12 C5-Branched dibasic acid metabolism	4	3	0	1	0	2	2	1	0	1	1	20
1.13 Inositol metabolism	6	2	0	1	3	4	7	2	3	2	4	5
2. Energy metabolism												
2.1 Oxidative phosphorylation	24	7	0	6	11	11	33	10	14	9	13	14
2.2 ATP synthesis	8	2	0	3	3	1	11	4	3	4	1	1
2.4 Carbon fixation	11	3	0	3	5	11	11	3	5	3	13	23
2.5 Reductive carboxylate cycle (CO2 fixation)	12	7	0	1	4	8	9	2	4	3	7	13
2.6 Methane metabolism	6	4	0	0	2	5	6	0	4	2	6	26
2.7 Nitrogen metabolism	11	2	0	5	4	14	12	5	5	2	15	64
2.8 Sulfur metabolism	5	1	0	1	3	9	6	3	1	2	9	30
3. Lipid metabolism												
3.1 Fatty acid biosynthesis (path 1)	6	2	0	3	1	11	7	3	3	1	6	14
3.2 Fatty acid biosynthesis (path 2)	8	2	0	5	1	6	6	3	2	1	5	8
3.3 Fatty acid metabolism	14	6	1	6	1	17	21	13	7	1	16	28
3.4 Synthesis and degradation of ketone bodies	2	0	0	1	1	2	8	4	3	1	3	6
3.5 Sterol biosynthesis	4	1	1	2	0	4	4	2	2	0	9	35
3.6 Bile acid biosynthesis	11	7	1	2	1	11	11	6	4	1	10	27
3.8 Androgen and estrogen metabolism	7	5	0	2	0	9	7	4	3	0	8	26
4. Nucleotide metabolism												
4.1 Purine metabolism	27	11	0	11	5	28	32	14	11	7	32	99
4.2 Pyrimidine metabolism	16	8	1	5	2	15	22	9	12	1	22	61
4.3 Nucleotide sugars metabolism	6	4	0	0	2	3	4	2	2	0	4	30
5. Amino acid metabolism												
5.1 Glutamate metabolism	11	3	0	5	3	14	16	8	7	1	18	36
5.2 Alanine and aspartate metabolism	14	1	0	8	5	15	14	5	6	3	15	38
5.3 Glycine, serine and threonine metabolism	19	7	0	9	3	14	21	8	10	3	24	56
5.4 Methionine metabolism	6	1	0	4	1	9	6	5	1	0	8	24
5.5 Cysteine metabolism	8	2	0	2	4	11	7	5	2	0	9	23
5.6 Valine, leucine and isoleucine degradation	16	3	1	8	4	16	22	11	6	5	18	32
5.7 Valine, leucine and isoleucine biosynthesis	7	1	0	4	2	7	9	6	2	1	8	15
5.8 Lysine biosynthesis	11	1	0	6	4	10	9	4	3	2	8	31
5.9 Lysine degradation	19	8	0	8	3	14	19	12	6	1	17	47
5.10 Arginine and proline metabolism	18	4	0	8	6	20	22	11	7	4	20	71
5.11 Histidine metabolism	10	4	0	4	2	8	10	5	4	1	8	39
5.12 Tyrosine metabolism	18	8	0	7	3	19	19	11	5	3	19	67
5.13 Phenylalanine metabolism	13	5	0	3	5	11	14	7	6	1	12	40
5.14 Tryptophan metabolism	17	8	0	8	1	15	22	16	4	2	18	61
5.15 Phenylalanine, tyrosine and tryptophan biosynthesis	5	1	0	2	2	6	4	2	0	2	7	31
5.16 Urea cycle and metabolism of amino groups	10	1	0	5	4	14	10	2	6	2	11	35
6. Metabolism of other amino acids												
6.1 beta-Alanine metabolism	14	4	1	7	2	13	11	8	1	2	10	32
6.2 Taurine and hypotaurine metabolism	1	0	0	0	1	1	2	2	0	0	3	14
6.3 Aminophosphonate metabolism	4	0	0	3	1	3	5	2	3	0	5	15
6.4 Selenoamino acid metabolism	7	0	0	4	3	12	12	6	4	2	15	22
6.5 Cyanoamino acid metabolism	2	1	0	1	0	1	7	5	1	1	6	19
6.6 D-Glutamine and D-glutamate metabolism	2	0	0	1	1	2	2	1	0	1	2	12
6.7 D-Arginine and D-ornithine metabolism	3	1	0	0	2	2	3	0	2	1	2	10
6.9 Glutathione metabolism	5	1	0	0	4	4	9	5	3	1	6	27
7. Metabolism of complex carbohydrates												
7.1 Starch and sucrose metabolism	18	2	0	13	3	18	20	13	6	1	20	75
7.2 N-Glycans biosynthesis	7	4	0	1	2	7	7	2	4	1	9	27
7.3 O-Glycans biosynthesis	3	1	0	1	1	2	6	5	1	0	3	8
7.5 Aminosugars metabolism	6	3	0	2	1	6	10	5	5	0	10	39
7.8 Glycosaminoglycan degradation	1	1	0	0	0	1	1	1	0	0	1	13
7.9 Chondroitin / Heparan sulfate biosynthesis	5	3	0	1	1	4	6	2	4	0	4	18
7.10 Keratan sulfate biosynthesis	1	0	0	1	0	1	2	1	1	0	1	6
8. Metabolism og complex lipids												
8.1 Glycerolipid metabolism	24	10	0	9	5	22	25	10	13	2	22	80
8.3 Inositol phosphate metabolism	8	4	0	4	0	4	8	5	2	1	3	25
8.4 Sphingophospholipid biosynthesis	1	1	0	0	0	1	2	1	1	0	2	8
8.5 Phospholipid degradation	3	2	0	0	1	3	1	1	0	0	1	11
8.6 Sphingoglycolipid metabolism	11	1	1	9	0	7	10	8	2	0	4	20
8.9 Globoside metabolism	2	1	0	1	0	2	2	1	1	0	1	12
8.11 Prostaglandin and leukotriene metabolism	8	2	0	1	5	8	7	4	3	0	6	19
9. Metabolism of cofactors and vitamins												
9.2 Riboflavin metabolism	4	1	0	3	0	2	2	2	0	0	2	13
9.3 Vitamin B6 metabolism	6	4	0	1	1	3	6	4	1	1	5	23
9.4 Nicotinate and nicotinamide metabolism	11	2	0	7	2	7	15	8	7	0	7	32
9.5 Pantothenate and CoA biosynthesis	8	2	0	4	2	9	8	6	2	0	7	27
9.7 Folate biosynthesis	5	2	1	0	2	5	6	1	4	1	5	25
9.8 One carbon pool by folate	5	3	0	2	0	10	7	3	3	1	8	24
9.10 Porphyrin and chlorophyll metabolism	18	4	0	10	4	12	27	15	8	4	13	56
9.11 Ubiquinone biosynthesis	19	10	0	5	4	13	28	11	14	3	14	22
10. Biosynthesis of secondary metabolites												
10.1 Terpenoid biosynthesis	0	0	0	0	0	0	2	0	2	0	4	12
10.3 Flavonoids, stilbene and lignin biosynthesis	6	3	0	1	2	7	8	5	3	0	7	39
10.4 Alkaloid biosynthesis I	5	2	0	3	0	6	3	3	0	0	5	36
10.8 Streptomycin biosynthesis	2	0	0	2	0	3	4	2	1	1	4	14
10.9 Erythromycin biosynthesis	2	0	0	2	0	3	3	2	1	0	3	6
11. Biodegradation of xenobiotics												
11.4 Nitrobenzene degradation	4	1	0	3	0	5	4	2	1	1	3	17
11.9 Tetrachloroethene degradation	6	4	0	1	1	3	2	2	0	0	3	5
11.10 Styrene degradation	4	2	0	2	0	3	6	5	0	1	5	18
11.1 gamma-Hexachlorocyclohexane degradation	6	3	0	3	0	5	5	3	2	0	4	12
11.1 Fluorene degradation	3	1	0	2	0	4	2	2	0	0	2	13
11.2 Benzoate degradation via CoA ligation	22	8	1	10	3	18	25	14	10	1	18	38
11.2 Benzoate degradation via hydroxylation	7	2	0	5	0	7	5	4	1	0	5	45

^a^*A. caninum *– 839 multiple and 786 unique mappings; *A. ceylanicum *– 957 multiple and 840 unique mappings. ^b ^Cluster, ^c ^Enzymes

### Homologs in other organisms, orthologs within *Ancylostoma spp*. and estimates of selective pressure

**Figure 3:**
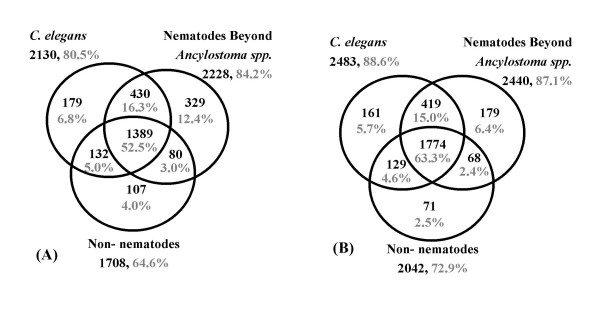
Venn diagram showing distribution of *A. caninum *(A) and *A. ceylanicum *(B) cluster BLAST matches by database. Amino acid level homologies (≥ e-05) were identified to non-*Ancylostoma *sequences for 65.8% (2,646/4,020) of *A. caninum *and 83.1% (2,801/3,369) of *A. ceylanicum *clusters. Databases used are: for *C. elegans*, Wormpep v.97 and mitochondrial protein sequences; for other nematodes, all GenBank nucleotide data for nematodes except *C. elegans *and *Ancylostoma*; for non-nematodes, nrGenBank (3/20/2003) with all nematode sequences removed.

**Figure 4:**
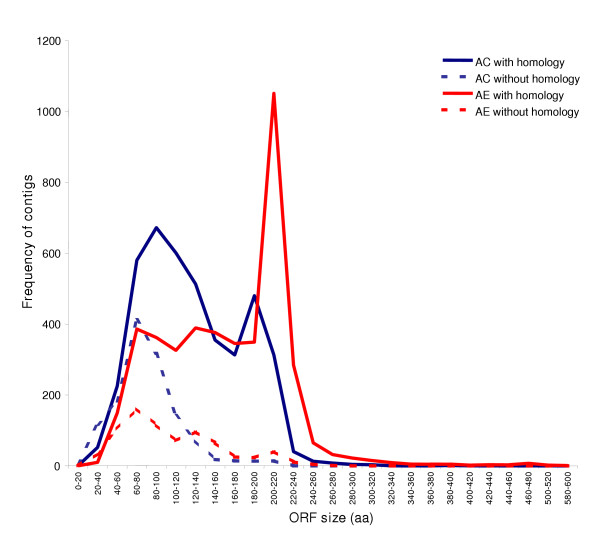
Distribution of *A. caninum *and *A. ceylanicum *contigs with and without database amino acid level homology by size of the longest predicted open reading frame (ORF).

Within *A. ceylanicum *clusters, 83% had homology to proteins from other organisms as compared to only 66% for *A. caninum *(Figure 3). To investigate why contigs from closely related species would show a difference in identified homologies, we compared sequence lengths and the open reading frame (ORF) lengths of contigs with and without homologies in both species. EST lengths and contig lengths, respectively, were shorter for *A. caninum *(410 and 549 nucleotides) than for *A. ceylanicum *(490 and 637 nucleotides). The differences were even more striking for ORFs (Figure 4). Hence, *A. caninum *contigs very likely identify fewer homologs because these sequences are shorter, contain smaller ORFs, and probably include more 3' UTR versus the superior quality dataset from *A. ceylanicum*. Most likely, differences in library construction and sampling rather than intrinsic differences between the species explain this discrepancy. Accounting for such differences is important as it keeps analysis focused upon interesting features of the dataset related to the organisms' biology rather than artifactual differences arising from data collection.

**Figure 5:**
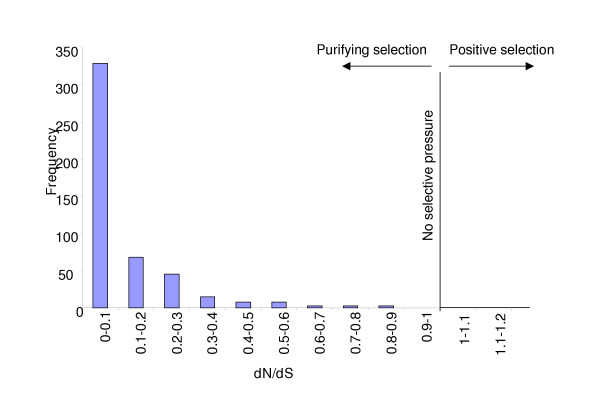
Distribution of dN/dS ratios among *Ancylostoma *ortholog pairs. dN and dS are the rates of nonsynonymous and synonymous amino acid substitutions, respectively.

The distribution of identified homologs (Figure 3) was consistent with earlier observations [17]. Besides *C. elegans*, one of the more informative nematode datasets for this study is a collection of 4,780 ESTs from the human hookworm *Necator americanus *to which homologies were commonly found (42% and 38% respectively). Within *Ancylostoma *itself homologies were common with 34% of total *A. caninum *clusters matching the *A. ceylanicum *dataset and 44% of total *A. ceylanicum *clusters matching *A. caninum*. Searching for putative orthologs between all *A. caninum *and *A. ceylanicum *contigs resulted in 1,304 reciprocal best TBLASTX hits. The ortholog pair members were very similar in GC composition (46% and 47%) and the average length of alignment was 327 bp. All ortholog pairs (574) were under purifying selection (dN/dS < 1; Figure 5) and the average dS was 0.65 ± 0.83 and dN was 0.11 ± 0.2. The average dN/dS ratio (~0.17) is higher than that reported for *C. briggsae/C. elegans *(~0.06; [18]), and closer to that for mouse/human (0.115; [24]), indicating that the levels of purifying selection are somewhat different. In addition, to examine if this purifying selection is more frequently detected in genes with essential function, we cross-referenced the *C. elegans *genes matched by *Ancylostoma *orthologs with a list of *C. elegans *genes with available RNA interference (RNAi) information (; eg. [25]). Of the 67% of the orthologous genes matching *C. elegans *genes with available RNAi data, 45% had an observable phenotype after transcript knock-down. A vast majority of the observed phenotypes were severe (88% sterility and embryonic lethality). *Ancylostoma *orthologs matching *C. elegans *genes that had phenotypes showed a somewhat lower dN/dS ratio than those matching genes that remained wild type after RNAi treatment, though the difference was not statistically significant at P < 0.05 (0.09 vs. 0.14; sign. diff. at P < 0.2).

**Figure 6:**
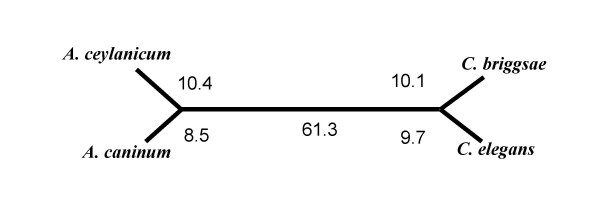
Relative distance based upon protein maximum likelihood. *A. ceylanicum *to *A. cananum *distance is similar to *C. elegans *to *C. briggsae *distance. *Ancylostoma *to *Caenorhabditis *distance for any species is 4.3X the *Ancylostoma *to *Ancylostoma *distance and 4.1X the *Caenorhabditis *to *Caenorhabditis *distance. The length of each line segment is proportional to the calculated branch length between the species.

In a 4-way comparison of orthologs in *C. elegans, C. briggsae, A. caninum*, and *A. ceylanicum*, the phylogenetic distance between *A. caninum *and *A. ceylanicum *is almost identical to that between *C. elegans *and *C. briggsae *and the distance between the two genera is just over four times the within genera distance (Figure 6). Average branch lengths for the set of 452 orthologous proteins did not show a significant difference in relative rate of molecular change. Maximum likelihood trees [26] were constructed for each 4-way ortholog and relative branch lengths compared between *Ancylostoma spp*. for both the protein and nucleotide sequences. For protein sequences, 109 trees had equal branch lengths for the hookworm species while 175 trees had longer *A. caninum *branches and 168 had longer *A. ceylanicum *branches. To look for differences between genes in the *Ancylostoma *species, we constructed a distribution of branch length differences between each of the sister species pairs in our tree. Because some genes may be rapidly evolving in all nematode lineages we evaluated a subset of the trees where the difference in branch lengths between *C. briggsae *and *C. elegans *were less than one standard deviation from the mean but which had significantly different branch lengths in the two *Ancylostoma *species. This final dataset had 23 genes with significantly longer branch lengths in *A. ceylanicum *and 16 in *A. caninum *(1 SD, P < 0.05). However the set did not show any significant bias towards either species (p < 0.34, sign test). This suggests there is no significant rate difference in protein evolution between the two hookworms, although some of these genes are relatively rapidly evolving. Repeating the analysis for nucleotide sequences we find marginally significant differences (p < 0.08; sign test).

### Using the *C. elegans *genome to interpret hookworm sequences

As expected, the *C. elegans *genome provides the best source of information for interpreting hookworm sequences as a majority of *A. caninum *and *A*. *ceylanicum *clusters with BLAST homologies outside *Ancylostoma *had *C. elegans *homologs (Figure 3; 25 most conserved nematode genes between each *Ancylostoma *species and *C. elegans *are available online; [see Additional file 2]). Furthermore, *C. elegans *orthologs of hookworm genes with available RNAi or other data provide information that may be relevant to understanding the role of the parasite genes. Of all the *Ancylostoma *clusters with *C. elegans *homology, 97% and 92% matched *C. elegans *genes with available RNA interference knock-down information , and in turn 33% and 37% of these *C. elegans *genes produce RNAi phenotypes (versus a rate of only ~15% phenotypes for all *C. elegans *genes). Phenotype classification [see Additional file 3] showed that *C. elegans *genes with expressed *Ancylostoma *homologs were somewhat more likely to have severe phenotypes [17]. Hence, certain genes in the *Ancylostoma *datasets encode proteins if disabled may disrupt survival of the parasite. Some examples include abundant clusters (AC00023.cl, AE01104.cl; Table 5 and Table 6). A group of particular interest is proteins that are required for nematode survival and lack strong homologies outside of the phyla (nematode-specific), since these targets could provide for nematode control without toxicities to the host or other non-target organisms. Of the *Ancylostoma *nematode-specific clusters (Figure 3), 85 and 91 respectively had *C. elegans *matches with RNAi phenotypes. Among these, AC04398.cl and AE00474.cl matched hypothetical protein F42A8.1 (1e-65, 2e-76 respectively), a gene without a mammalian homolog yet likely involved in multiple developmental processes based on observed mutant phenotypes [25]. Homologs are found in at least 13 nematode species to date including free-living species (3), and parasites of mammals (8) and plants (1). Further analysis will identify additional genes which warrant detailed investigation.

**Table 5:** The most abundantly represented transcripts in the *A. caninum *cDNA libraries

					non-redundant GenBank			
							
*A. caninum *cluster id	ESTs per cluster	ESTs from library				Accession		*C. elegans *gene
		iL3	taL3	ssL3	Best identity descriptor	SW / TR^a^	E-value	Wormpep97^b^
AC00932.cl	203	186	0	17	*Ancylostoma duodenale *cytochrome oxidase subunit I	CAD10437	2e – 304	C06G4.2b
AC00471.cl	120	2	0	118	*C. elegans *CMP-sialic acid transporter	O02345	2e – 83	ZK896.9^b^
AC00048.cl	114	100	11	3	*C. elegans *hsp-12.6 alpha-B-crystallin	Q20165	6e – 40	F38E11.2^b^
AC01032.cl	104	104	0	0	*Ancylostoma duodenale *cytochrome oxidase subunit II	AAL50814	1e – 09	-
AC01031.cl	93	90	0	3	*Ancylostoma duodenale *cytochrome oxidase subunit III	CAD10435	2e – 143	-
AC00807.cl	69	4	2	63	*Necator americanus *ancylostoma secreted protein 1 precursor	AAD13340	3e – 28	F33A8.2
AC00205.cl	65	51	1	13	*Ancylostoma duodenale *COX2, cytochrome c oxidase subunit II	NP_579953	4e – 125	F26E4.12
AC00967.cl	54	9	0	45	*Ancylostoma ceylanicum *cathepsin D-like aspartic protease	AAO22152	8e – 174	R12H7.2
AC00134.cl	44	44	0	0	*C. elegans *putative protein, nematode specific	NP_497272	9e – 53	K02F3.9^b^
AC01029.cl	39	37	2	0	*C. elegans *stress associated endoplasmic reticulum protein	NP_510604	9e – 37	F59F4.2^b^
AC00137.cl	38	37	0	1	*C. elegans *RNA recognition motif	CAB03222	4e – 35	R06C1.4^b^
AC00023.cl	38	36	0	2	*C. elegans *rpl-2 Ribosomal Proteins L2	Q9XVF7	3e – 148	B0250.1^b^
AC00060.cl	36	36	0	0	*Ancylostoma caninum *secreted protein ASP-2 precursor	AAC35986	2e – 134	F11C7.3b
AC01400.cl	32	1	0	31	*C. elegans *ham-2 zinc finger protein	NP_508781	1e – 20	C07A12.1^b^
AC00976.cl	32	29	0	3	*Tetrahymena pigmentosa *metallothionein MT-2	AAL87687	6e – 12	K11G9.6
AC00193.cl	31	7	0	24	*Pisum sativum *putative senescence-associated protein	BAB33421	2e – 61	F58H1.7
AC00931.cl	29	6	0	23	novel	-	-	-
AC00079.cl	28	25	2	1	*Ostertagia ostertagi *unknown protein	AAC08432	8e – 06	-
AC00980.cl	27	11	0	16	*C. elegans *Glycerol kinase	AAA79749	8e – 70	R11F4.1^b^
AC00971.cl	26	9	0	17	*C. elegans *rpl-1 Ribosomal Protein Large subunit	NP_491061	5e – 116	Y71F9AL.13a^b^
AC01023.cl	25	22	2	1	*Ostertagia ostertagi *putative ES protein F7	CAD20464	9e – 87	F02A9.2
AC00913.cl	25	6	17	2	*C. elegans *ribosomal protein L37	O62388	2e – 50	W01D2.1^b^
AC02930.cl	24	0	0	24	*C. elegans *calponin-like protein	NP_504712	4e – 119	T25F10.6^b^
AC00252.cl	24	12	12	0	*C. elegans *rpl-43	CAB54440	4e – 49	Y48B6A.2^b^
AC01020.cl	23	20	3	0	*C. elegans rps-15 *Ribosomal Protein Small subunit RPS-15	NP_492384	1e – 88	F36A2.6^b^

^a ^SW/TR is Swiss-prot and TrEMBL Proteinknowledgebase .

^b ^*C. elegans *homolog has higher probability match than the best GenBank descriptor.

**Table 6:** The most abundantly represented transcripts in the *A. ceylanicum *cDNA libraries

				non-redundant GenBank			
						
*A. ceylanicum *cluster id	ESTs Per cluster	ESTs from Library			Accession		*C. elegans *gene
		iL3	Ad	Best identity descriptor	SW / TR^a^	E-value	Wormpep97^b^
AE00908.cl	323	320	3	*C. elegans *stress associated endoplasmic reticulum protein	NP_510604	9e – 37	F59F4.2^b^
AE00787.cl	205	205	0	*C. elegans *hsp-12.6 alpha-B-crystallin	Q20165	7e – 39	F38E11.2^b^
AE01104.cl	155	155	0	*C. elegans *microsomal signal peptidase 25 kDa subunit	Q9XWW1	8e – 80	Y37D8A.10^b^
AE00463.cl	119	118	1	*C. elegans *dlc-1 dynein light chain (10.3 kD)	NP_498422	9e – 56	T26A5.9^b^
AE00121.cl	110	0	110	*C. elegans *vit-3 Vitellogenin 3 precursor	NP_508613	7e – 121	F59D8.1^b^
AE00890.cl	84	84	0	*C. elegans *spp-4 SaPosin-like Protein family	NP_509237	5e – 16	T08A9.8^b^
AE00065.cl	84	84	0	*C. elegans *putative endoplasmic reticulum protein	NP_508656	1e – 35	F47B7.1^b^
AE00360.cl	74	74	0	novel	-	-	-
AE00048.cl	72	72	0	*C. elegans *rpl-29 60S ribosomal protein L29	NP_502671	6e – 25	B0513.3^b^
AE00003.cl	70	3	67	novel	-	-	-
AE01410.cl	63	60	3	*Ostertagia ostertagi *putative ES protein F7	CAD20464	3e – 86	F02A9.2
AE00056.cl	62	56	6	*C. elegans *hypothetical protein	AAK77617	5e – 39	M01H9.3a^b^
AE00464.cl	61	61	0	novel	-	-	-
AE00227.cl	59	0	59	*Zea mays *extensin-like protein	S49915	2e – 31	ZK84.1
AE00746.cl	54	0	54	*C. elegans *protein contains chitin binding peritrophin-A domain	AAA19083	3e – 54	B0280.5^b^
AE00750.cl	50	36	14	*C. elegans *far-7 fatty Acid/Retinol binding protein	NP_493708	7e – 45	K01A2.2a^b^
AE00072.cl	47	0	47	*Beta vulgaris *chitinase	S51939	6e – 12	C34D4.11
AE00591.cl	45	45	0	*C. elegans *hypothetical protein	AAF99918	7e – 26	F29B9.11^b^
AE01221.cl	44	44	0	*Volvox carteri *hydroxyproline-rich glycoprotein DZ-HRGP	CAB62280	1e – 29	Y59A8B.19
AE01407.cl	41	41	0	*C. elegans *elt-3 GATA-binding transcription factor like	CAA93510	5e – 18	K02B9.4^b^
AE00033.cl	41	0	41	*Nippostrongylus brasiliensis *hsp-20 Nbhsp20	CAA50655	8e – 56	T27E4.3
AE01361.cl	40	39	1	*C. elegans *ICD-1 inhibitor of cell death	AAA68776	1e – 75	C56C10.8^b^
AE00322.cl	39	37	2	*C. elegans *hypothetical protein	CAB54416	7e – 28	Y38E10A.24^b^
AE00536.cl	36	34	2	*Homo sapiens *unnamed protein product	BAB71316	4e – 134	F25B5.4a
AE00503.cl	35	13	22	*C. elegans *eft-3 elongation factor 1-alpha	NP_498520	3e – 283	F31E3.5^b^

^a ^SW/TR is Swiss-prot and TrEMBL Proteinknowledgebase .

^b ^*C. elegans *homolog has higher probability match than the best GenBank descriptor.

Repeating the analysis in Stein et al. [18] indicates that 6–7% of *C. elegans *and *C. briggsae *proteins are candidate "orphans", lacking homologs outside of the species. We examined whether these genes are truly orphans that have arisen in a *Caenorhabditis *sub-lineage or are instead genes present in an ancestral nematode that have been lost or evolved beyond recognition in one species. Of candidate orphan proteins, ten from *C. elegans *(Table 7) and 27 from *C. briggsae *[see Additional file 4] match *A. caninum *and/or *A. ceylanicum *clusters, with three and eight, respectively, having matches in both species. Most of the *C. elegans *orphans are hypothetical proteins of unknown function though some had functional information from InterPro domains (R10E9.3) or mutant phenotypes (ZK686.1). Therefore, at least a portion of the genes identified in either *C. elegans *or *C. briggsae *as "orphans" are actually ancestral nematode genes with homologs found in other clade V species and further clade V sequencing will likely reveal more such cases.

**Table 7:** *C. elegans *candidate orphans (1,358 out of 21,437) matching *Ancylostoma *clusters

*C. elegans *gene^a^	Descriptor	*Ancylostoma *cluster id^b^	ESTs *per *cluster	E-value	C. elegans *gene *length (aa)	Matched region length (%)	%ID
F31E8.1	Hypothetical protein	AC05087.cl	1	1e – 07	249	14.5	45
F57B10.14	Hypothetical protein	AE02023.cl	2	3e – 20	56	75.0	69
R10E9.3	Contains Cytochrome C heme-binding site	AC02329.cl	1	2e – 10	149	79.2	32
		AE00556.cl	2	5e – 19	149	97.3	31
T07A9.13	Putative nuclear encoded protein	AE02236.cl	1	4e – 31	111	91.9	49
Y35H6.1	Hypothetical protein	AE03902.cl	1	6e – 23	161	47.2	48
Y41C4A.3	Hypothetical protein	AE00269.cl	14	1e – 05	162	49.4	37
Y54G2A.27	Hypothetical protein	AC04390.cl	1	2e – 05	229	14.4	38
		AE01938.cl	9	8e – 07	229	11.4	48
ZC487.3	Hypothetical protein	AC04655.cl	1	2e – 08	79	81.0	38
ZK686.1	Nuclear transition protein	AC00867.cl	16	2e – 07	44	68.2	62
		AE01651.cl	3	3e – 07	44	68.2	62
ZK84.5	Hypothetical protein	AC00410.cl	3	6e – 11	84	70.2	46

^a ^Of 21,437 proteins 1,358 were candidate orphans

^b ^AC, *Ancylostoma caninum; *AE, *A. ceylanicum*

### Abundant transcripts expressed in *Ancylostoma *species

The 25 most abundantly represented clusters account for 14% and 19% of ESTs for *A. caninum *and *A. ceylanicum *respectively. The representation of the abundant transcripts varied from shared to stage-specific (Table 5 and Table 6). Hookworm developmental stages differ in habitat, morphology and behavior, hence highly represented gene transcripts may identify functions that are important to the parasites at various stages. Differences in gene expression between *A. ceylanicum *stages have been demonstrated previously for several genes [27,28]. Our comparison of iL3 and adult suggests additional examples (see Discussion). In fact, only 9% of the *A. ceylanicum *clusters are shared between iL3 and adult (Figure 2) and of the 25 largest clusters, 23 were biased toward one of the developmental stages (Table 6). While representation in EST collections generally correlates with source expression level [29], artifacts can occur [30,31]. Differences in expression are most likely to be accurate when comparing the most abundant transcripts in each stage. Therefore, while follow-up work is needed to confirm expression levels, examination of ESTs provides a list of candidates for various expression profiles.

## Discussion

### Overview

We have taken a genomics approach to the study of hookworm species, key parasites of humans and domestic animals that are related to the model nematode *Caenorhabditis elegans*. Nearly 20,000 ESTs from *Ancylostoma caninum *and *A. ceylanicum *identified approximately 7,000 genes including over 1,300 likely orthologs represented in both species. Close to 900 genes encode putative enzymes involved in 88 metabolic pathways. Over 3,100 genes contain recognizable protein domains many of which have been categorized in the Gene Ontology hierarchy. 4,600 genes have homologs in *C. elegans *including numerous nematode-specific genes and hundreds with observable RNAi phenotypes. ESTs originated from libraries representing infective L3 larva, stimulated L3, tissue arrested L3, and adults resulting in an improved rate of gene discovery and allowing the identification of transcripts abundant in various stages.

### Gene expression in iL3 and dauers

Infective L3 (iL3) are developmentally-arrested, non-feeding pre-parasitic stages, which when triggered by the infection process and host-specific signals reactivate, molt and complete development. A similar stage in *C. elegans *is called the dauer larva. In *Ancylostoma *species host factors such as dog serum stimulate feeding and an activation response in serum stimulated L3 (ssL3) [32] that approximates the transition to parasitism in the host [33]. *A. caninum *tissue-arrested L3 (taL3) recovered from infected mice are a distinct population that potentially share properties with the arrested iL3. Developmentally arrested, non-feeding larvae would be expected to be dependent on stored energy reserves and lipid metabolic pathways; accordingly, the KEGG biochemical pathway mappings show a substantive number of clusters for fatty acid metabolism especially with the *A. ceylanicum *iL3 clusters (Table 4).

*C. elegans *microarray experiments identified 540 dauer-enriched genes along with genes involved in dauer-recovery [34]. *C. elegans *SAGE experiments identified 358 candidate dauer-specific genes [35]. Genes shown to be abundantly expressed in *C. elegans *dauers include a variety of genes that may play roles in extended survival including heat shock protein encoding genes like *hsp-12.6 *and *daf-21 *(Hsp90), *ctl-1 *(cytosolic catalase), *sod-3 *(superoxide dismutase), and *hil-1 *and *hil-3 *(Histone H1's). A number of genes identified both in *Ancylostoma *L3s and *C. elegans *dauers are discussed below.

#### Heat-shock Proteins

hsp-12.6, a member of the *hsp-20 *family, was one of the most highly represented clusters in *A. caninum *iL3 and taL3 as well as *A. ceylanicum *iL3 (Table 5 and Table 6). Among a *Strongyloides stercoralis *EST collection, the gene is also found in iL3 but not L1 [17]. *C. elegans *hsp-12.6 is upregulated in dauer and starved L1s [34,36] and is a transcriptional target of the FOXO transcription factor DAF-16 [37]. Unlike other HSPs, *C. elegans hsp-12.6 *is not stress-induced and does not prevent aggregation of unfolded proteins, suggesting a novel role. AE00033.cl, found exclusively in adult ESTs, encodes an ortholog of the *Nippostrongylus brasiliensis *HSP-20 protein. *Nb-hsp-20 *is more similar to the HSP-16 group of the HSP-20 family of small HSPs in *C. elegans*, is also expressed in the adult [38], and is not stress regulated, suggesting that it may function as an adult version of *hsp-12.6*.

#### Candidate stress-response proteins

*A. caninum *iL3 showed abundant clusters encoding homologs of the mitochondrial cytochrome oxidase subunits I, II, III and a stress associated endoplasmic reticulum protein not seen in ssL3 (Table 5). One *A. ceylanicum *iL3 abundant cluster encoded a ribosome-associated membrane 4 protein (RAMP4) involved in ER protein translocation [39] which is over-expressed in hypoxia and suppresses degradation of ER membrane proteins [40]. A homolog of *C. elegans spp-4 *was also expressed at high levels in *A. ceylanicum *iL3. *spp-4 *encodes an amoebapore, a member of the saposin-like protein superfamily that kill bacteria by forming membrane ion channels [41]. Amoebapore proteins are one of a number of putative stress response proteins regulated by DAF-16 in *C. elegans *[37,42]. These proteins, also including lysozyme and thaumatin, may provide a defense against worm pathogens and contribute to dauer longevity [43]. Hookworm free-living stages are also soil dwelling microbiverous organisms exposed to soil pathogens, so it is possible that *spp-4 *plays an antibacterial role in *A. ceylanicum*.

### Gene expression in ssL3, adults, and multiple stages

In contrast to iL3, *A. caninum *ssL3 showed a CMP-sialic acid transporter, cathepsin D-like aspartic protease, calponin-like protein, and ham-2 zinc finger protein among the abundant transcripts. While the significance of these molecules is unknown, upregulation of an aspartic protease during the transition to parasitism and tissue penetration/migration is consistent with its role in degradation of serum proteins and collagens [44].

Abundant adult-specific clusters are likely to be involved in reproduction. For example, *A. ceylanicum *(Table 6) encodes an ortholog of the *C. elegans *VIT-3 protein, a lipid binding protein and major yolk component [45]. VIT-3 is expressed exclusively in the *C. elegans *adult hermaphrodite intestine, secreted, and taken up by oocytes. Two clusters encode genes involved in metabolism of chitin, an important constituent of the nematode eggshell [46]. One encodes a protein similar to *C. elegans *protein C34D4.11, and shows some similarity to a beet chitinase; the other encodes an ortholog of *C. elegans *B0280.5, a protein required for early embryonic development [47]. B0280.5 mRNA is expressed specifically in the adult hermaphrodite germ line and is a target of GLD-1, an RNA binding protein required for oocyte meiotic cell cycle progression [48].

#### ASP's

While there are differences in the cluster profiles among *Ancylostoma *stages, there are shared transcripts as well. For example, the *Ancylostoma *secreted protein ASP-1 and ASP-like cDNAs are present in abundance in both *A. caninum *iL3 and ssL3. The secretion of ASP-1 protein by ssL3s was noted as a marker of the transition to parasitism [12]. These results support conclusions made by Wang and Kim [34] that arrested larvae are transcriptionally prepared for dauer exit and upon receipt of appropriate stimulatory signals, exit from the arrested state is accompanied by a burst of translational activity in addition to further transcriptional activity. In contrast to ASP-1, ESTs for ASP-2 were exclusively detected in *A. caninum *iL3.

#### FAR Proteins

Two of the most abundant *A. ceylanicum *transcripts encode fatty acid/retinol binding (FAR) proteins (Table 6). FAR proteins are novel fatty acid and retinol binding proteins described in nematodes including *A. caninum*, other Strongylida, filarial, and plant parasitic species [49-51]. In *C. elegans *8 FAR members are divided into 3 groups. All the parasitic nematodes FARs described to date are most similar to the *C. elegans *A group containing *Ce-far-1*, -*2*, and -*6*. Seven *A. ceylanicum *clusters encode FAR proteins. Four of which (9 ESTs) were found in the adult cDNA library; clusters AE00748.cl and AE03203.cl were nearly identical to *Ac-far-1 *and *Ac-far-2 *(98% and 99% nucleotide identity) whereas cluster AE02490.cl showed the highest similarity to *Ce-far-1 *and AE01700.cl to *Ac-far-2*. The iL3 specific clusters AE01410.cl (60/63 ESTs from iL3) and AE03983.cl (2/2 ESTs from iL3) were both most closely related to a FAR protein from *Ostertagia ostertagi *[52], and more distantly to *Ac-far-1 *and *-2*. Therefore, as seen in other parasitic nematodes, most *A. ceylanicum *FAR proteins are related to *C. elegans *group A FAR proteins. However, the *A. ceylanicum *cluster AE00750 was most similar to group C FAR protein *Ce-far-7*. Group C proteins differ from the other FARs in important ways including lacking an N-terminal signal peptide (suggesting an intracellular location), containing several cysteines, and failing to bind DAUDA [53]. AE00750.cl represents the first report of a FAR-7 like protein from a parasitic nematode. The function of FAR proteins is unknown but may represent a lipid acquisition system in which released FARs bind to lipids followed by uptake of the complex by a specific receptor mediated process. Retinoids are required for nematode growth and development, but are not synthesized by the worms. In parasitic nematodes, release of FAR proteins may also modify local inflammatory and immunological responses by delivering or sequestering biochemically important lipids [54].

## Conclusion

The application of genomic approaches to hookworms has resulted in more than a 100-fold increase in available sequence data from *Ancylostoma *species thereby allowing an initial bioinformatic analysis of transcripts from these important parasites and establishing a foundation for the eventual completion of a hookworm genome. Semi-automated informatic approaches that are now being applied to all nematode sequences [55] allow uniform comparisons across many genomes and provide databases for further exploration. Transcripts in *A. caninum *and *A. ceylanicum *include clear candidates for stage specific expression representing the very different biological processes underway in different points of the lifecycle. The availability of the *C. elegans *and *C. briggsae *genomes has allowed highly informative comparisons to the two hookworm species showing extensive overlap in gene complements, including genes demonstrated to be essential in *C. elegans *and numerous genes specific to nematodes. As the most closely related major human pathogen to *C. elegans*, hookworms provide an attractive near-term application for using a model organism to better understand and eventually control a key disease-causing species. Beyond categorization of hookworm genes, clear research avenues are available to apply this information to improved methods for hookworm control including anthelmintic and vaccine development, diagnostics, population studies, as well as better understanding of fundamental aspects of hookworm biology, such as host immune system modulation.

## Methods

### Nematode extraction

A Shanghai strain of *A. caninum *was maintained in beagles as described [56]. Infective L3 (iL3) were recovered from 7–10 day old coprocultures using a modified Baermann technique, washed clean of debris with BU buffer (50 mM Na_2_PO_4_/22 mM KH_2_PO_4_/70 mM NaCl, pH 6.8; [57]), and snap-frozen by immersion in liquid N_2_. Frozen larvae were stored at -80°C until used for library construction. Serum stimulated larvae (ssL3) were generated by incubating iL3s harvested from a North Carolina strain of *A. caninum *in 5% normal dog serum for 20–24 h at 37°C, 5% carbon dioxide. Tissue-arrested L3 larvae (taL3) were recovered from BALB/c mice infected with 1,000–1,500 iL3 (North Carolina strain) and euthanized at 10–14 days post-infection [58].

A Warsaw strain of *A. ceylanicum *was maintained in Syrian hamsters as described [59], and L_3 _recovered, washed, and frozen as above. For the recovery of adult stage *A. ceylanicum*, infected hamsters with patent infections were sacrificed and the small intestine removed. The intestine was cut into 3 sections, opened longitudinally, and hung in 50 ml centrifuge tubes containing phosphate buffered saline at 37°C for 2–3 hrs. Following incubation, adult worms were recovered from the sediment, washed free of debris, and snap-frozen as above. All animals were housed and treated in accordance with institutional care and use committee guidelines.

### Preparation of *A. caninum *staged RNA and cDNA libraries

Pulverization for the ssL3 and taL3 was performed using an Alloy Tool Steel Set (Fisher Scientific International). Total RNA from adult and larval parasites was prepared using TRIzol Reagent (GibcoBRL, Life Technologies or Invitrogen, Carlsbad, CA).

SMART based serum stimulated L3 library – Library construction was based on the SMART cDNA library construction system (Clontech Laboratories; [60]). mRNA was extracted from 10 μg of total RNA using a Dynabeads mRNA Purification kit (Dynal Biotech) with some modifications. First strand synthesis was performed with the mRNA bound to the oligo-dT of the Dynabeads using Superscript II RT (Invitrogen, Life Technologies) and the primer smart T7_3G_5. Concatemers were digested with Not I on the bead. Second strand synthesis was performed with the smartT7_5 and the smartCDSII primer. Amplification of the cDNA was performed with the smartT7_5 and smartT7_3 primers with cycling parameters of 95°C for 5 minutes, seven cycles of 95°C for 5 seconds, 60°C for 5 seconds and 68°C for 6 minutes followed by a 4 minute extension at 68°C. Following amplification, the cDNA was purified using the High Pure PCR Product Purification Kit (Roche). The final 5 cycles of PCR introduced the deoxy-UMP primers needed for cloning into the pAMP1 vector (Invitrogen, Life Technologies). cDNA fragments >1 kb were size selected on a 0.8% TAE agarose gel and cloned into the pAMP1 vector following the CloneAMP pAMP1 System (Invitrogen, Life Technologies). The ligation mix introduced into *E. coli *DH10B chemically competent cells (GibcoBRL, Life Technologies) resulted in 4.36 × 10^5 ^primary transformants.

SL1-PCR-based tissue arrested L3 library – mRNA was extracted from 2 μg of total RNA using a Dynabeads mRNA Purification kit (Dynal Biotech) and eluted with 10 μl 10 mM Tris-HCl. First strand synthesis was performed using linker primer (GAGAGAGAGAGAGAGAGAGAACTAGTCTCGAGTTTTTTTTTTTTT) and Superscript II RT (Invitrogen, Life Technologies). Amplification with Taq Polymerase used the SL1 (GGGTTTAATTACCCAAGTTTGA) and Xhop (GAGAGAGAACTAGTCTCGA) primers and 5 μl of the first strand reaction. Cycling parameters were 95°C for 5 minutes, 30 cycles of 95°C for 1 minute, 47°C for 1 minute, 72°C for 3 minutes followed by 5 minutes at 72°C. cDNA fragments >1 kb were size selected on a 0.8% TAE agarose gel and cloned into the pCRII-TOPO vector following the TOPO TA protocol (Invitrogen). The ligation mix was introduced into *E. coli *DH10B chemically competent cells (GibcoBRL, Life Technologies).

Hawdon infective L_3 _library – Frozen *A. caninum *L3 pellets were ground to powder on a mortar pre-chilled with liquid nitrogen. Total RNA was isolated from the powder using Trizol reagent (Invitrogen, Carlsbad, CA). Poly (A)+ RNA was isolated from total RNA using the Oligotex mRNA isolation kit (Qiagen, Chatsworth, CA). Approximately 5 μg of mRNA was used to construct a directional cDNA library in Lambda ZAP II (Stratagene, La Jolla, CA) as previously described [61]. pBluescript phagemid were mass excised prior to sequencing. The library had >95% recombinants, and insert size varied from 700–3,000 bp. The library was amplified once (10^6 ^pfu).

### Preparation of *A. ceylanicum* staged RNA and cDNA libraries

Hawdon adult and infective L3 *A. ceylanicum *libraries – Total RNA and poly (A)+ mRNA were isolated from the appropriate *A. ceylanicum *life-cycle stage using Trizol reagent and the Oligotex mRNA isolation kit as described above. Approximately 5 μg of mRNA was used to construct directional cDNA libraries in Lambda ZAP II (Stratagene, La Jolla, CA) as previously described [61]. Both libraries had 99% recombinants with inserts ranging from 500–2500 bp (average 1500 bp), and each underwent one round of amplification (10^6 ^pfu). Inserts were mass excised as described above.

SL1-PCR-based infective L3 and adult libraries – cDNA was PCR amplified, using SL1-EcoRI primer on the 5' end and oligo(dT)-XhoI on 3' end, gel fractionated [62], and non-directionally cloned into pCR-TOPO-XL (Invitrogen), following XL-Topo TA cloning protocol. The cDNA inserts were excised with EcoRI.

### Sequencing and clustering

Sequencing, EST processing and clustering were performed as described [17]. Information for clone requests and sequence trace files are available at . The completed cluster assemblies, NemaGene *Ancylostoma caninum *v 2.0 and *A. ceylanicum *v 2.0, were used as the basis for all subsequent analyses and are available for searching and acquisition by FTP at . "Fragmentation", defined as the representation of a single gene by multiple non-overlapping clusters, was estimated by examining *Ancylostoma *clusters with homology to *C. elegans *[17]. Overall representation of *Ancylostoma *genes is based on a theoretical gene number of 21,437, comparable to *C. elegans *wormpep97.

### Analysis and functional assignments

Homology assignments – WU-BLAST sequence comparisons [63,64] were performed using *A. caninum *and *A. ceylanicum *contig consensus sequences which were further organized into clusters. Consensus sequences were used to search multiple databases, including the non-redundant GenBank (3/20/2003) and Wormpep v.97 *C. elegans *(Wellcome Trust Sanger Institute, unpublished) protein databases. Internally constructed databases using intersections of data from Genbank, allowed examination of sequences in specific phylogenetic distributions. Homologies were reported for E (expect) value scores of ≥ 1e -05.

To identify cases where *Ancylostoma *homologs in *C. elegans *have been surveyed for knock-down phenotypes using RNA interference, Wormpep BLAST matches were cross-referenced to a list of 17,042 *C. elegans *genes with available RNAi information (20^th ^February 2005) . For each *Ancylostoma *cluster, only the best *C. elegans *match was considered.

Functional classification – Clusters were assigned putative functional categorization using two methods. First, InterProScan v.3.1  was used to search contig translations versus InterPro domains (11/08/02) [65,66]. Using InterPro, clusters were mapped to the three organizing principles of the Gene Ontology (GO_200211_assocdb.sql) [67]. Mappings are stored by MySQL database, displayed using AmiGo (11/25/02) , and are available at . Second, clusters were assigned by enzyme commission number to metabolic pathways using the Kyoto Encyclopedia of Genes and Genomes (KEGG) database (2/24/2004)[68]. All matches better than 1e-10 were taken into consideration.

Orthologs and dN/dS ratio – *A. caninum */ *A. ceylanicum *orthologs were determined by reciprocal best TBLASTX match using a threshold of E value ≥ 10^-5^. In addition, the ORFs accepted to be the correct translation were required to have the best *C. elegans *gene match in the same frame as the TBLASTX matches. Only continuous alignments longer than 30 amino acids were accepted. 'Suboptimal alignment program' (Jason Stajich, unpublished), scripted using tools in Bioperl [69] and utilizing 'yn00' from PAML [70], calculated the synonymous (dS) and non-synonymous substitutions (dN) per ortholog pair. A 4-way orthologs were assigned by using SSEARCH [71,72] to find the best *C. elegans *and *C. briggsae *homolog for each ortholog pair of *A. caninum *and *A. ceylanicum*. Orthologs were assigned if both sequences agreed on the best hits. Multiple sequence alignments were performed with MUSCLE [73]. Trees were built using the programs 'protml' and 'nucml' for protein and nucleotide sequences respectively [26]. An exhaustive search was used first to enumerate the possible topologies and then -R rearrangement search was used to identify the most likely branch lengths and bootstrap values. Only genes for which well supported topologies where *A. caninum *and *A. ceylanicum *appeared as sisters were used in subsequent analysis. Tree branch lengths were parsed and processed with Perl scripts written using modules from the Bioperl package and statistical tests were applied with the R package [74].

## List of abbreviations used

Ad, adult parasite stage; BLAST, basic local alignment search tool; dN, non-synonymous substitutions; dS, synonymous substitutions; EST, expression sequence tag; GO, gene ontology; iL3, infective third larval stage; KEGG, Kyoto encyclopedia of genes and genomes; PCR, polymerase chain reaction; ssL3, serum-stimulated L3; taL3, tissue-arrested L3.

## Authors' contributions

MM, JPM, SWC, RKW, and RHW conceived and designed the research plan and participated in all aspects of data collection and analysis. MM, JPM, MD, TW, JX, and JES analyzed and interpreted the data. PA, JH, and WK contributed material and constructed cDNA libraries. MM, JPM, PA, and JH drafted the manuscript. All authors read and approved the final manuscript.

## Supplementary Material

Additional File 1 Accession numbers. Click here for file

Additional File 2 Most conserved nematode genes between *A. caninum *and *C. elegans*. Click here for file

Additional File 3 Classification of *C. elegans *RNAi phenotypes for genes with *A. caninum *and *A. ceylanicum *homologs. Click here for file

Additional File 4 *C. briggsae *candidate orphans matching *Ancylostoma *clusters. Click here for file
