# Supplementary material for: Investigating hookworm genomes by comparative analysis of two Ancylostoma species
Source: BMC Genomics. 2005 Apr 26;6:58. doi: 10.1186/1471-2164-6-58 (PMC1112591; doi:10.1186/1471-2164-6-58)
Supplement: Additional File 1 — Accession numbers. [file 1471-2164-6-58-S1.doc]

**Additional file 1:**

Nucleotide sequences are available from GenBank, EMBL, and DDJB under the accession numbers for *A. caninum*: AW181253 - AW181857, AW588187 - AW588597, AW589037 - AW589049, AW589122 - AW589196, AW626753 - AW627336, AW700205 - AW700931, AW734988 - AW735502, AW782961 - AW782980, AW870290 - AW870656, BE352341 - BE352565, BF249526 - BF250089, BF250121 - BF250923, BG232148 - BG232753, BG438297 - BG438375, BI744214 - BI744515, BI773248 - BI773332, BM077299 - BM077991, BM129848 - BM130432, BM285291 - BM285359, BQ125044 - BQ125340, BQ666050 - BQ667755, BI704582 and *A. ceylanicum*: BI704890, BM130433 - BM131232, BQ274663 - BQ276243, BQ288062 - BQ289786, BU780919 - BU781047, CA033060 - CA033334, CA341190 - CA341541, CA408081 - CA408219, CB174715 - CB176577, CB189579 - CB190870, CB275505 - CB277332, CB338884 - CB339180. The sequences are also available at <http://nematode.net/>.
